# Do We Need Psychological Outcome Measures in the Management of Lateral Elbow Tendinopathy (LET)?

**DOI:** 10.3390/jcm11195916

**Published:** 2022-10-07

**Authors:** Dimitrios Stasinopoulos

**Affiliations:** Department of Physiotherapy, Faculty of Health and Caring Sciences, University of West Attica, Agiou Spyridonos 28, Egaleo, 12243 Athens, Greece; dstasinopoulos@uniwa.gr

Many outcome measures have been developed in the evaluation of LET. The recommended outcome measures for LET assessment according to the Bateman et al. study [1] are:

1. The Patient Rated Tennis Elbow Evaluation for Disability
2. Time off work,
3. Pain-free grip strength and
4. a Numerical Rating Scale measuring pain on gripping.

All the above outcome measures should also be used until future studies recommend alternative, more robust, measures of participation in life activities, physical function capacity, and pain on activity/load [1].

However, I wonder if psychological factors such as kinesiophobia, depression, stress, and catastrophization can affect the management of LET. Their influence on, or association with, lower limb tendinopathies is beginning to emerge in the literature [2]. These associations have led to the suggestion that sensitization of the nervous system, and impaired pain processing, may explain persistent tendinopathy pain states, and ongoing loss of function that can occur following tissue-based intervention in tendinopathy [3].

I believe that the above psychological factors can affect LET rehabilitation and that researchers should find ways to assess these factors. Researchers can assess the psychological factors using:

5. The Hospital Anxiety and Depression Scale (HADS) to detect and measure the severity of depression and anxiety
6. The Tampa Scale for Kinesiophobia (TSK) to measure fear of movement
7. The Life Orientation Test-Revised (LOT-R) to measure dispositional optimism or pessimism.

On the other hand, future studies should investigate the psychometric properties of these instruments for the LET population. Further research is needed to indicate which of the above scales is more representative of the LET condition. Finally, if the above-recommended scales are not appropriate for LET patients, new validated measures should be developed.

Overall, these additional outcome measures may assist in the identification of factors that predict chronicity and poor treatment results in LET patients.
